# Event and Comment

**Published:** 1915-04

**Authors:** 


DENTAL REGISTER

Vol. LXIX APRIL 1915 No. 4

### EVENT AND COMMENT.

COPPER CEMENTS. We are reprinting in this issue two articles each treating a phase of the cement filling problem of great interest and consequence to the dentist. One on the bacteriological action of the chief copper cements in use by the profession of to-day. The article is long and a good deal technical, but as it was published in a scientific journal not seen by the dentists, we republish it that it may be available to such as may be interested in the subject. We are not wholly prepared to accept all the conclusions of the author of this paper, as there are some things to be said from the practitioners' experiences that do not seem to harmonize exactly with the scientific findings recorded. Yet it is such a carefully prepared article, and apparently such a frank statement that it should serve to stimulate further research by other scientists, and be given the final provings of the practitioners who, after all, are to render the verdict. The question as to which particular make of copper cement possesses the better inhibiting properties will take care of itself in due time; but it should be a matter of great concern, not only to the manufacturers of cements, as well as to the dental practitioners, that we know exactly how copper cements when set into comparatively solid masses inclosed in tooth tissue become efficient as antiseptics without losing their integrity to an extent that will destroy their efficiency as filling materials. We can not see that the author makes this point as clear as may be necessary to enable us to depend upon it in practice.

The second paper on the chemistry of cements is well worth considering, as it most clearly sets forth some facts about cement that every dentist ought to know that he may get the best service out of them. If we knew, as this article informs us, the nature of cements and the method of combining in the setting process, we should take better care in the mixing of our cements and have more satisfactory results as to working qualities and durability. The paragraph on the next to the last page in regard to the manner of incorporating the powder with the liquid ought to be copied and posted at the top of the mixing slab until it is clearly fixed in the dentist's memory and until he shall have formed a proper habit of making the mix. We trust that every reader of this journal will read carefully both of these articles.

---

THE GREATEST PROBLEM OF THE DAY IN DENTISTRY. Under this caption we are reprinting an editorial from the editor of *Items of Interest* in this issue. There may be some question in our readers' minds as to whether this problem as stated by Dr. Ottolengui is as grave as he seems to believe. There are some tremendous problems still to be solved by our profession, and this may be one of them, and, like the author, we do not know the solution to this one any more than many another practitioner. We suppose some time or other it will get a satisfactory solution. It may be that the time will come, even in our day, that we shall have no poor people with diseased pulps. Or it may be that some one will lessen the cost of the roentgenogram so that the poor may have one at a nominal cost. Isn't it more than likely that we shall in the future solve this problem by careful aseptic root canal surgery combined with rational therapeusis? It certainly is expecting a great deal of the average dentist to insist that he subject his patients to the X-ray exposure both before and after root canal treatments. Thorough cleansing of canals by present day well-approved methods and practicable closure with adaptable materials that have a tendency to inhibit infection has met with reasonable success for many years. Now that our attention has been called to the fact that careless, or, some wrong use of these methods have resulted disastrously to the welfare of some patients, it does not seem necessary that we should get discouraged or attempt the impracticable. We do not wish to be understood as trying to discourage progress. On the contrary, we are in cordial sympathy with the work of Rhein, Callahan and Ottolengui, as well as all others who are trying to find a way out of this difficulty. At present we see no better way than every honest and conscientious practitioner is following. The serious difficulty with this present idea is that of cost. It is no more serious here than are the same problems in the inlay operations, the crown and bridge operations, painless dentistry, dental prosthesis, etc. So far as we can see now, our reply must be simliar to the answers we have been giving to all our problems for all time: Do the best we know or can under each and all circumstances.

